# Transcriptome Analysis of Human Peripheral Blood Mononuclear Cells Exposed to Lassa Virus and to the Attenuated Mopeia/Lassa Reassortant 29 (ML29), a Vaccine Candidate

**DOI:** 10.1371/journal.pntd.0002406

**Published:** 2013-09-12

**Authors:** Juan Carlos Zapata, Ricardo Carrion, Jean L. Patterson, Oswald Crasta, Yan Zhang, Sachin Mani, Marti Jett, Bhawna Poonia, Mahmoud Djavani, David M. White, Igor S. Lukashevich, Maria S. Salvato

**Affiliations:** 1 Institute of Human Virology, University of Maryland School of Medicine, Baltimore, Maryland, United States of America; 2 Texas Biomedical Research Institute, San Antonio, Texas, United States of America; 3 Virginia Bioinformatics Institute at Virginia Tech, Blacksburg, Virginia, United States of America; 4 Walter Reed Army Institute of Research, Silver Spring, Maryland, United States of America; 5 Center for Disease Control and Prevention, Atlanta, Georgia, United States of America; University of Texas Medical Branch, United States of America

## Abstract

Lassa virus (LASV) is the causative agent of Lassa Fever and is responsible for several hundred thousand infections and thousands of deaths annually in West Africa. LASV and the non-pathogenic Mopeia virus (MOPV) are both rodent-borne African arenaviruses. A live attenuated reassortant of MOPV and LASV, designated ML29, protects rodents and primates from LASV challenge and appears to be more attenuated than MOPV. To gain better insight into LASV-induced pathology and mechanism of attenuation we performed gene expression profiling in human peripheral blood mononuclear cells (PBMC) exposed to LASV and the vaccine candidate ML29. PBMC from healthy human subjects were exposed to either LASV or ML29. Although most PBMC are non-permissive for virus replication, they remain susceptible to signal transduction by virus particles. Total RNA was extracted and global gene expression was evaluated during the first 24 hours using high-density microarrays. Results were validated using RT-PCR, flow cytometry and ELISA. LASV and ML29 elicited differential expression of interferon-stimulated genes (ISG), as well as genes involved in apoptosis, NF-kB signaling and the coagulation pathways. These genes could eventually serve as biomarkers to predict disease outcomes. The remarkable differential expression of thrombomodulin, a key regulator of inflammation and coagulation, suggests its involvement with vascular abnormalities and mortality in Lassa fever disease.

## Author Summary

The virulent Lassa fever virus (LASV) and the non-pathogenic Mopeia virus (MOPV) infect rodents and, incidentally, people in West Africa. The mechanism of LASV damage in human beings is unclear. There is no licensed Lassa fever vaccine and therapeutic intervention is usually too late. The ML29 vaccine candidate derived from Lassa and Mopeia viruses protects rodents and primates from Lassa fever disease. Peripheral blood mononuclear cells from healthy human subjects were exposed to either LASV or ML29 in order to identify early cellular responses that could be attributed to the difference in virulence between the two viruses. Differential expression of interferon-stimulated genes as well as coagulation-related genes could lead to an explanation for Lassa fever pathogenesis and indicate protective treatments for Lassa fever disease.

## Introduction

Lassa Fever (LF) is one of the most neglected tropical diseases and has the second highest global incidence of any viral hemorrhagic fever (VHF), after Dengue HF. The estimated “at risk” population in Sierra Leone, Guinea, and Nigeria, may be as high as 59 million. The number of symptomatic cases per year is around 300,000 with between 5,000 and 10,000 deaths [1] [2] [3]. LF is also one of the most prominent imported VHF affecting public health of non-endemic countries worldwide and has been classified as a category A bio-threat agent [4], [5], [2], [6].

Lassa virus (LASV) belongs to the *Arenaviridae*, a fast-growing family of rodent-borne viruses, currently including 22 enveloped viruses with bi-segmented, ambisense single-stranded RNA genomes divided into two groups [7], [8]. The Old World (OW) group contains the prototypic lymphocytic choriomeningitis virus (LCMV) which can cause neuropathology in adults, fetal abnormalities in newborns, and a fatal LF-like disease in immunocompromised patients. OW viruses also include LASV; and non-pathogenic viruses, Mopeia (MOPV), Mobala (MOBV), and Ippy (IPPYV), as well as newly-found LuJo (pathogenic) [9] and Luna (unknown pathogenicity) [10] viruses. The New World group includes Junín, Machupo, Guanarito, Sabia, and Chapare viruses associated with severe VHF in South America [11].

The disease mechanism for LF is still not clearly understood. As with other VHF, events that determine LF fatal outcome occur very early in the disease and depend on the host immune system [12], [13]. Macrophages (MP) and dendritic cells (DC) are the initial targets of infection, followed by viremic seeding of a wide variety of cells and organs. High viremia and elevated levels of AST in plasma are two major risk factors predicting fatal outcome [14]. Although the most consistent pathologic finding is hepatocellular necrosis, the extent of liver damage is usually insufficient to implicate hepatic failure as the cause of death [14], [2]. Functional damage of platelets and endothelial cells seems to play an important role [15], [16], [2]. However, contribution of pro- and anti-inflammatory stimuli to LF pathogenesis is not clearly understood.

Our laboratory [17], [18] and others [19], [20], [21], [22] showed that LASV infection is associated with suppression of pro-inflammatory responses contributing to unchecked viremia and fatal outcome. Study of LF pathogenesis is complicated by the absence of reliable and validated animal models. Using a surrogate model of LF in rhesus macaques inoculated with the WE strain of LCMV [23] we showed that early transcriptional changes in blood, mostly in genes involved in complement, interferon, and pro-inflammatory pathways, can discriminate virulent from non-virulent infection [24].

There is no approved LF vaccine and therapeutic options are limited to intravenous or oral ribavirin, which is often impractical in endemic areas. Vaccine candidate ML29 is a reassortant containing the L genomic segment, or replication genes, of MOPV and the S segment encoding the major structural proteins of LASV [25], [26]. Eighteen mutations distinguish the ML29 genome from the parental arenaviruses and likely contribute to ML29 attenuation [27]. In animal models, ML29 is broadly cross-protective against diverse strains of LASV [28], and was able to elicit LASV-specific immunity in a monkey model for AIDS [29], [30].

In the current study we have directly exposed human PBMC from healthy donors to LASV or to its derivative, ML29. We used PBMC profiling previously to show that ML29 elicited less inflammatory gene expression than MOPV [29], recapitulating similar profiles of ML29 in naïve primates [26], and in SIV-infected macaques [30]. Human PBMC exposed to virus is a model for the viremic stage of infection *in vivo* when much of the transcriptome response is due to non-infectious signal transduction by viral particles [31] [24]. PBMC were used here and in other studies [32] [33] [34] rather than cell lines [35] [36] to more closely simulate the situation *in vivo* in which circulating blood cells are exposed to virus-infected sites. Here we have shown that *in vitro* PBMC exposure to arenaviruses with different pathogenic potential, LASV and ML29, resulted in differential expression of ISG, apoptotic, NF-kB, and coagulation pathway genes.

## Materials and Methods

### Viruses and cells

LASV (strain Josiah) was obtained from CDC (Atlanta, GA) and ML29 was previously described [25], [26], [27].

Vero E6 cells (ATCC, CRL-1586) were cultured in Dulbecco's modified Eagle's medium (DMEM, GIBCO-BRL) supplemented with 10% fetal bovine serum (FBS, GIBCO-BRL), 1% penicillin-streptomycin, and L-glutamine (2 mM); incubated at 37°C in 5% CO_2_ for 24 hours. Vero cell monolayers were infected with LASV and ML29 at a multiplicity of infection (MOI) of 0.01 and incubated 1 hour at 37°C in 5% CO_2_, washed with PBS and then covered in DMEM 2% FBS. Supernatants were collected at 48 and 72 hours post infection, titrated in Vero cells and stored at −70°C at 10^7^ PFU/ml.

Human PBMC were obtained by apheresis from healthy volunteers followed by Ficoll-hypaque isolation [37] and resuspended in RPMI 1640 medium with 10% heat-inactivated human AB serum (Sigma).

Monocytes were isolated from purified PBMC by plastic adherence and immature dendritic cells (DC) were generated from them after 5 days culture in supplemented RPMI. Briefly, the method for generating DC was to plate human PBMC at 1.67×10^7^ cells/2 ml/well in a 6-well plate in RPMI, 10% fetal calf serum. After 2 hrs, non-adherent cells were removed and the adherent monocytes were fed with 2.5 ml RPMI containing 20 ng/ml GM-CSF (Peprotech, Rocky Hill NJ), and 20 ng/ml IL-4 (Peprotech). After 5 days of incubation, cells were harvested, analyzed by flow cytometry, and processed for incubation with virus. On average, 22% of the cells were differentiated, monocyte-derived DC (DR+, CD11c+). [38].

### Ethics statement

Human blood samples were used under an IRB exemption to Dr. Salvato for the use of de-identified human cells obtained through the Red Cross.

### Human PBMC and DC exposure

1.2×10^7^ PBMC from healthy donors were exposed to 1 MOI of LASV or ML29 (BSL-4 facilities at SFBR, San Antonio, TX) for 45 minutes at 37°C in 5% CO_2_ and plated in 6 well plates in duplicate. Supernatants and cells were harvested at 4, 8, and 24 hours post-exposure. Cells were re-suspended in Trizol and kept at −70°C for RNA isolation.

1×10^7^ DC from 3 healthy donors were exposed to 1 MOI of LASV, ML29 or LPS (as an inhibitor control: *LPS is usually a positive control, here it was used to inhibit thrombomodulin*) for 45 minutes at 37°C in 5% CO_2_ (BSL-4 facilities at CDC, Atlanta, GA). Supernatants and cells were harvested at 24 hours post-exposure. Supernatants were kept at −70°C for ELISA analysis.

### Gene expression profiling using cDNA microarrays

Total RNA was isolated from PMBC samples using the Trizol method (Invitrogen, Carlsbad, CA) followed by a cleaning step with Rneasy mini kit (Qiagen, Valencia, CA), according to the manufacturer's instructions. Quality and quantity of all RNA samples were evaluated on the Agilent 2100 BioAnalyzer 116 system (Agilent Technologies, Palo Alto, CA) by looking at 18 & 28 s rRNA peaks and by the RIN (RNA integrity number). High quality RNA was labeled and hybridized according to Affymetrix protocols using the GeneChip human genome U133 Plus 2.0 array (Affymetrix, Santa Clara, CA) and as described previously [24]. This chip covers the whole human genome using 54,000 probe sets representing approximately 22,000 genes.

### Microarray data analysis

Images from each hybridization, were inspected manually and percentage of present calls of each array was checked. Cluster [39] and Principal Component Analyses (PCA) [40] against all conditions using genes with normalized maximum value/minimum value >5 were also performed. Raw data from the arrays were normalized at probe level using a robust multichip average of G+C content algorithm (gcRMA) [41], [42] and then log2 transformed. The detection calls (Present, Marginal, Absent) for each probeset were obtained using Affymetrix GeneChip Operating Software (GCOS) (http://www.affymetrix.com/browse/products.jsp?productId=131429&navMode=34000&navAction=jump&aId=productsNav#1_1). Only genes with at least one present call across all compared hybridizations were selected for further statistical analysis. All data have been submitted under series record GSE41300.

For the identification of differentially-expressed genes the Linear Models for Microarray Data (LIMMA) package was used [43].

Due to high variation among donors the pairwise comparison Significance Analysis of Microarrays (SAM) was used to eliminate disturbance. PBMC from each donor were compared before and after exposure at different time points.

To identify the over-represented Gene Ontology (GO) terms in each list of differentially expressed genes from 3 donors and 2 repetitions, the GO::TermFinder was used [44]. GO terms with adjusted p value (false discovery rate or FDR) less than 0.05 were selected.

In order to identify over-represented Pathways from each list of differentially expressed genes from 3 donors and 2 repetitions, the Kegg's pathway [with adjusted p value (FDR) less than 0.05 for each list of genes] was used.

### Validation of cDNA array data by quantitative RT-PCR

Due to the high number of interferon-related genes involved in the LASV and ML29 gene profiles, they were selected for validation by RT-PCR. 24 hour post-exposure (hpe) RNA samples, from 3 donors, and 3 time points used in the microarray test, were evaluated by the Interferon and Receptor PCR Array Kit (Human Interferon and Receptor array, Cat# No. PAHS-064, SABiosciences, Frederick, MD). This platform tested 84 interferon-related genes and 5 housekeeping genes for data normalization. Briefly, 1 µg of RNA from PBMC was reverse-transcribed in a 20-µL reaction volume into first-strand cDNA using SuperArray's ReactionReady First Strand cDNA Synthesis Kit (Cat. No C-01, SABiosciences) containing random and oligo dT primers. After mixing the cDNA with RT2 SYBR Green/ROX qPCR Master Mix (Cat. No PA-012, SABiosciences), 10 µL of the cDNA mixture was dispensed into a 96 well format plate RT^2^ profiler PCR array (Cat. No PAHS-064, SAbioscience).

Real-time PCR was performed on an ABI Fast 7900 Real-Time PCR System (Applied Biosystems, Foster City, CA) using the following cycling parameters: 10 min at 95°C (heat activation step); 40 cycles of 15 sec at 95°C, 1 min at 60°C. Relative changes in gene expression were calculated using the ΔΔC_t_ (threshold cycle) method. This method first subtracts the ct (threshold cycle number) of the gene-average ct of the 5 house keeping genes on the array to normalize to the RNA amounts. Finally the ΔΔct is calculated which involves subtracting the normalized average ct of LASV samples -normalized average ct of the Control Samples. Then this ΔΔct is raised to the negative power of 2 in order to calculate the fold changes. The p value was calculated using a 2-tailed Student's *t* test. These results were treated to SAM data analysis to avoid donor variation.

### Flow cytometry and ELISA analysis of thrombomodulin expression

Whole blood from healthy donors was collected in heparin tubes, mixed with 20 ng/ml lipopolysaccharide (LPS Cat No-tlrl-ekpls, InvivoGen, San Diego, CA) as down-regulation control for thrombomodulin [45], MEM as non-stimulated control and ML29 virus. Then this mix was brought to 3 ml with RPMI 10% FBS and incubated for 4, 8, or 24 hours. For each time-point cells were stained with phycoerytrhin-conjugated mouse anti-human thrombomodulin monoclonal antibody (PE-CD141, BD Biosciences, San Jose, CA) and fluorescein isothiocyanate mouse anti-human CD14+ antibody (FITC-CD14+ BD Biosciences, San Jose, CA). Cells were collected (10,000 events per sample) using a FACSCalibur cytometer (BD Biosciences) and the data were analyzed using FlowJo 8.8.4 software (Tree Star, San Carlos, CA). The monocyte population was gated and percentages of double positive cells (CD14+ and CD141+) were determined.

Dendritic cells were isolated from 3 different individuals for virus exposure and expression of soluble thrombomodulin. Immature DC were derived from human PBMC as described above in “Viruses and cells”.

Soluble thrombomodulin was quantified by ELISA (Human Thrombomodulin ELISA kit. Cat. No ab46509, Abcam, Cambridge, MA), in DC supernatants after 24 hour of exposure to LPS, ML29, LASV or LASV plus LPS. Plates were read at 450 nm using SpectraMax M2 ELISA reader (Molecular Devices Corporation, Sunnyvale, CA). The estimated amount of thrombomodulin was calculated using the Softmax pro 4.8 program (Molecular Devices Corporation, Sunnyvale, CA) and presented in picograms per ml.

## Results

### Exposure of human PBMC to LASV and ML29 results in different transcription profiles

In a previous transcriptome profiling study using a surrogate LF model in monkeys we showed that significant changes in PBMC gene expression occurred during pre-viremic days 1, 2, and 3 [24], clearly differentiating pathogenic versus non-pathogenic infections even before detectable virus replication. Clearly, a large part of the response to viruses is due to signal transduction of uninfected bystander cells.

In this study, PBMC from three healthy donors were exposed to LASV or ML29 viruses at 1 MOI in duplicate and were collected at 4, 8, and 24 hours post-exposure (hpe) for isolation of total RNA. cDNA was hybridized to Affymetrix chips covering the whole human genome. Cluster and Principal Component Analysis (PCA) showed that all samples clustered together by time (4, 8, and 24 hpe), by donor (3 healthy donors) and by treatment (unexposed, LASV-exposed, and ML29-exposed cells) (Figure S1). Since donor variation exceeded treatment variation, pairwise Significance Analysis of Microarrays (SAM) was used to compare RNA samples before and after exposure for each donor at different time points. Differentially-expressed genes were defined as those that had at least a >2-fold changes with respect to media controls.

**Figure 1:**
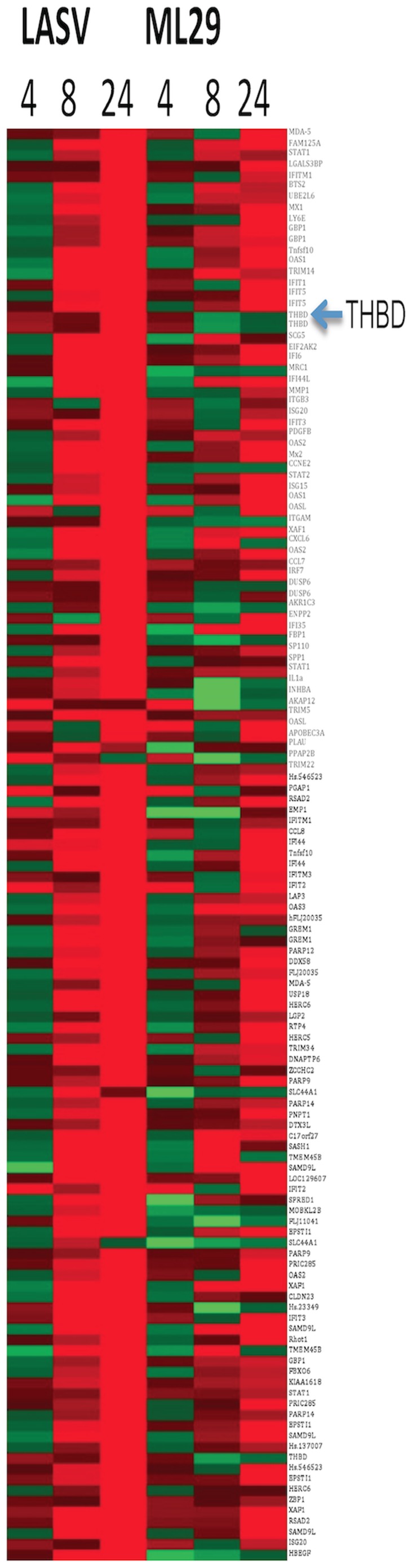
Heat map of the genes affected by ML29 and Lassa exposure in all three sets of human donor PBMC with respect to gene expression in non-exposed PBMC. RNA was extracted for analysis at 4, 8 and 24 hours after exposure. For a complete listing of all 122 genes, see Table S1 in the supplemental material. Green color represents down-regulated genes (two-fold or more when compared with non-exposed control). Red color represents up-regulated genes (two-fold or more when compared with non-exposed control).

A total of 122 genes were identified as being differentially expressed at tested time-points (Figure 1 and Table S1). Gene expression changes in ML29 and LASV-exposed PBMC were detected at 4 and 8 hpe respectively, with all genes up-regulated in LASV in contrast to ML29 with 11 down-regulated genes (4 at 4 hpe and 7 at 8 hpe). At 24 hpe LASV-treated PBMC showed the maximum changes (122 genes) in comparison with ML29-exposed samples.

### Gene ontology analysis of global changes in LASV versus ML29-exposed PBMC

**Figure 2:**
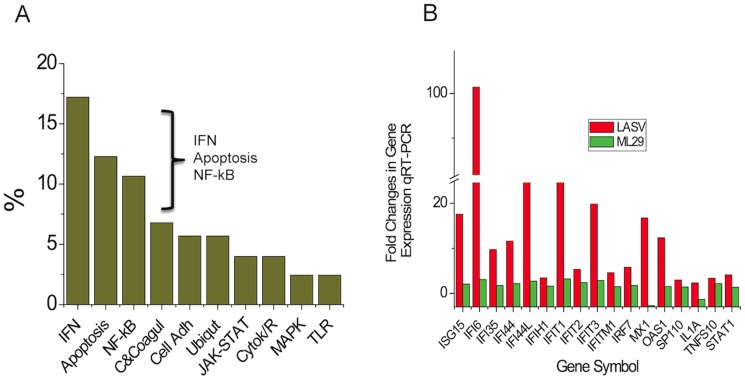
Categories of affected cellular processes. A) Kegg's pathway analysis showing the over-represented categories from each list of differentially-expressed genes after ML29 and LASV exposure for all time points and all donors. Interferon pathway genes had the highest percentage of presence (17.21%) followed by genes involved in apoptosis (12.29%) and NF-kappa B (10.65%). B) Fold changes in gene expression detected by quantitative RT-PCR.

In order to have a general view of the pathways affected by both virus exposures we performed gene ontology (GO) analysis of differentially expressed genes. The analyses revealed that the most affected genes were involved in type I IFN-stimulated pathways (21%–17 genes from 122), apoptosis (12.29%–15/122), and NF-kappa B pathways (10.65%–13/122) (Figure 2A). Although, Several genes could be put into more than one category, the interferon-related genes had the higher percentage of presence. From those categories the immune response, defense response and response to viruses were selected for validation analysis (Table 1). The most differentially expressed genes between LASV and ML29 are shown in Table 2.

**Table 1:** Validation of expression patterns of selected genes by quantitative RT-PCR.

GenBank accession no.	Gene symbol	Fold changes in gene expression DNA microarray		Fold changes in gene expression qRT-PCR	
		LASV	ML29	LASV	ML29
NM_005101	ISG15	28.95	14.82	17.58	2.09
NM_002038	IFI6	44.70	4.59	101.43	3.08
NM_005533	IFI35	16.78	6.38	9.75	1.76
NM_006417	IFI44	29.96/18.52^a^	20.31/9.24^a^	11.64	2.19
NM_006820	IFI44L	172.96	81.41	35.64	2.72
NM_022168	IFIH1	4.66/4.68^a^	4.69/2.92^a^	3.45	1.60
NM_001548	IFIT1	146.9	140.1	45.58	3.24
NM_001547	IFIT2	25.60/27.30^a^	35.30/25.25^a^	5.35	2.42
NM_001549	IFIT3	43.09	27.81	19.86	2.86
NM_003641	IFITM1	6.48/4.84^a^	2.48/1.91^a^	4.6	1.50
NM_001572	IRF7	7.51	2.92	5.81	1.75
NM_002462	MX1	34.45	17.98	16.80	−2.73
NM_002534	OAS1	28.90/39.28^a^	8.13/9.63^a^	12.39	1.56
NM_004509	SP110	6.33	2.48	2.99	1.43
NM_000575	IL1A	23.57	1.28	2.33	−1.31
NM_003810	TNFS10	6.18/5.33^a^	4.20/5.21^a^	3.38	2.21
NM_007315	STAT1	7.98/5.82^a^	2.22/2.11^a^	4.14	1.36

There was 76% correlation between microarray and qRT-PCR.

a Two different readings for a particular gene in the same chip.

**Table 2:** Genes most differentially-expressed between LASV and ML29.

GenBank accession no.	Gene symbol	Fold changes in gene expression DNA microarray					
		LASV			ML29		
		4 h	8 h	24 h	4 h	8 h	24 h
NM_002421	MMP1	−1.11	73.34	147.94	−1.18	−1.06	2.26
	RSAD2	1.29	8.20	136.84	1.52	1.45	94.02
NM_003020	SCG5	−1.13	17.82	33.95	−1.87	2.72	1.14
NM_080657	CXCL6	−1.31	30.69	26.29	−1.56	7.04	−1.34
NM_006273	CCL7	1.50	1.69	30.43	1.94	1.26	1.60
**NM_002438**	**MRC1**	**1.09**	**29.33**	**15.35**	**−2.06**	**−1.20**	**−1.34**
**AK001903**	**CDNA FLJ11041**	**1.26**	**2.95**	**23.77**	**−1.33**	**−3.80**	**−1.45**
**NM_002346**	**LY6E**	**1.26**	**2.13**	**23.77**	**−1.33**	**−3.80**	**−1.45**
NM_000575	IL1A	1.27	2.55	23.57	1.03	−4.16	−1.28
**NM_002192**	**INHBA**	**1.18**	**2.48**	**20.37**	**−1.55**	**−3.68**	**−1.07**
NM_006417	IFI44	2.58	2.74	18.52	−1.07	−1.12	9.24
NM_000361	THBD	1.71	1.26	14.56	1.28	−1.71	−1.07
NM_000582	SPP1	1.37	7.94	14.17	−1.24	1.01	1.14
NM_000212	ITGB3	1.55	−1.29	12.29	1.79	−1.39	1.48
NM_013372	GREM1	−1.43	9.96	8.89	−1.32	1.81	−1.00
NM_152594	SPRED1	−1.20	6.78	4.55	−5.10	1.84	1.18
NM_030776	ZBP1	1.25	1.06	6.24	1.66	1.04	1.81
NM_057749	CCNE2	−1.00	6.06	2.89	−1.18	−1.32	−1.28
NM_080546	SLC44A1	−1.32	3.33	1.26	−3.23	−1.14	−1.26
NM_033034	TRIM5	1.01	3.77	4.70	1.03	1.78	1.77
NM_194284	CLDN23	−1.38	4.58	3.57	−1.35	1.61	1.10
**NM_000632**	**ITGAM**	**1.04**	**1.16**	**4.36**	**−1.12**	**−1.49**	**−1.55**
NM_018381	Hypothetical protein FLJ11286	1.11	2.28	4.24	−1.11	1.65	1.77
**NM_001945**	**HBEGF**	**1.10**	**4.13**	**3.80**	**−2.45**	**−1.82**	**−1.11**
NM_024761	MOB1	−1.14	2.53	4.11	−1.83	−1.32	−1.07
**NM_000507**	**FBP1**	**1.22**	**1.08**	**3.74**	**−1.27**	**−2.15**	**−1.08**
**NM_003739**	**AKR1C3**	**−1.20**	**1.41**	**3.35**	**−1.37**	**−1.90**	**−1.24**
NP_005109.2	**TNFSF15**	1.62	3.31	**6.12**	1.21	**−4.22**	−1.15
NM_001946	DUSP6	1.17	1.17	3.17	1.26	−1.04	−1.02
NM_001423	EMP1	2.56	1.82	2.99	−3.59	**−3.12**	1.25

### LASV exposure up-regulates genes involved in innate immunity: IFN-stimulated genes, transcription factors, and genes involved in apoptosis

As shown in Figure 2A, the most striking differences involved gene expression in three different functional categories: IFN-stimulated, apoptosis, and NF-kB pathways, during the late time-point, 24 h after exposure.

To confirm the observed gene expression changes in PBMC exposed to LASV and ML29 viruses, the most affected probesets, the ISG, were selected for further validation. RNA samples from the 24-hr LASV- and ML29-exposed cells were assayed in duplicate using a human IFN and Receptors qRT-PCR Superarray kit (see Methods). Levels of expression of 17 gene-targets were expressed in fold changes (Table 1 and figure 2B).

Exposure of human PBMC to LASV resulted in over-expression of all 17 IFN-stimulated genes by 2- to 100-fold over background. Exposure of PBMC to ML29 had relatively little effect on the 17 tested genes. Comparative analyses showed a good correlation (76%) between the two techniques, DNA array and IFN qRT-PCR array (Table 1). For a few genes measured in the DNA array, ML29 exposure gave a higher background than quantitation by real-time PCR. This is because the housekeeping genes we used for normalizing the PCR data, including GAPDH, were not constantly expressed in the compared data sets, whereas probesets used for normalizing the microarray data varied less than 0.04% in all data sets (see Methods).

The most affected gene, IFI6, is the first IFN-stimulated gene known to inhibit apoptosis [46]. Among other most affected genes were IFI35, IFITM1, IRF-7, and SP110. While the function of the IFI35 gene is not well-defined, the IFITM proteins have roles in immune cell signaling and adhesion, cancer, germ cell physiology, and bone mineralization. IFITMs are also viral restriction factors that block entry of Influenza, Ebola, Dengue and West Nile viruses but not arenavirus-envelope pseudotyped particles [47] [48] [49]. IRF-7 is over-expressed in LASV-exposed PBMC and others found that it could be up-regulated in 3-day LASV-infected monocyte-derived macrophages [50]. IRF-7 is also known to limit the burst size of LCMV in culture but is not ultimately essential for LCMV clearance *in vivo* [51]. IRF-7 and IRF-3 are transcription factors which together with NF-kB and ATF-2/cJUN induce production of IFN-I. IRF-3 is required for the activation of IFN-β, which in turn, primes the expression of most IFN-α genes by IFN-induced IRF-7 through the STAT-1 pathway. Human SP110 plays an important role in resistance to intracellular pathogens such as human mycobacteria and intracellular cancers. Recently cellular proteins have been described that mediate the interaction of SP110 and viral proteins during viral infections [52].

Among apoptosis-related genes, the most striking difference was observed in the expression of matrix metalloproteinase, MMP-1 (interstitial collagenase), up-regulated >65-fold in LASV-exposed cells versus ML-29-exposed cells (Table 2). Proteins of the MMP family normally breakdown extracellular matrix during embryonic development, reproduction, and tissue remodeling, as well as in disease processes, such as arthritis and metastasis. MMP-1 is known to interact with Tat, the HIV transactivating protein, and this interaction results in the degradation of Tat and depression of Tat-mediated neurotoxicity and transactivation [53].

Two other apoptosis-related genes, SPP1 and INHBA, were up-regulated 14- and 20-fold, respectively (Table 2). SPP1, a secreted arginine-glycine-aspartate (RGD)-containing phosphoprotein 1 (previous names: osteopontin, bone sialoprotein I), enhances IFN-γ and IL-12 production and contributes to host defense, bone formation, and wound healing by stimulating macrophage migration as well as protecting against viral and bacterial infections through its pro-Th1 effect [54]. Inhibin-beta A (INHBA) joins Inhibin-alpha to form a pituitary follicle-stimulating hormone (FSH) secretion inhibitor. Inhibin has been shown to negatively-regulate gonadal stromal cell proliferation and to have tumor-suppressor activity. INHBA is also up-regulated by vaccinia and inhibits a diverse array of cytokines by enabling vaccinia virus E3 protein to antagonize several distinct but interlinked signaling cascades [55].

Exposure of human PBMC to LASV was associated with strong up-regulation (22-25-fold) of CXCL6 and IL1A genes in comparison to ML29 exposure (Table 2). Among CXC chemokines, CXCL6, a granulocyte chemotactic protein-2, shares the highest functional homology with IL-8. Both CXCL6 and IL1A exhibit disparate regulatory effects on recruited neutrophil responses [56] [57].

### Exposure and modulation of thrombomodulin expression

**Figure 3:**
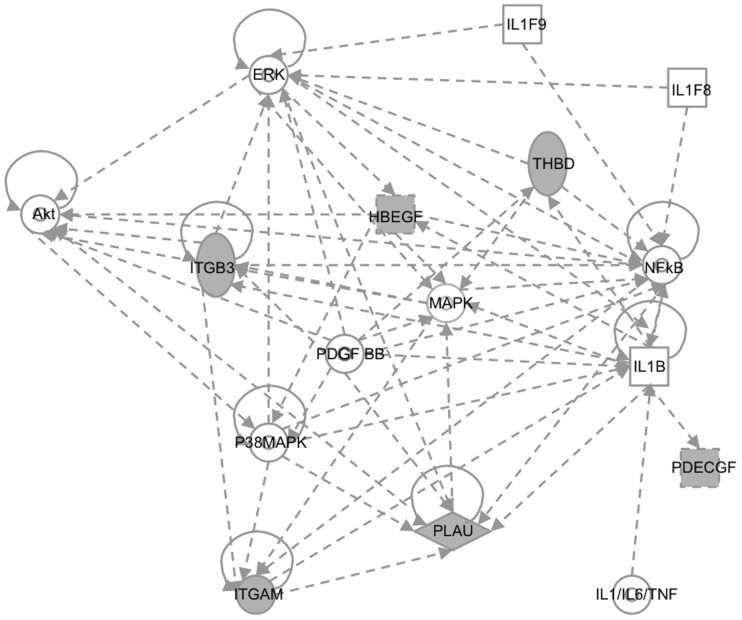
Gene Ontology (GO) analysis of coagulation-related genes. Gray figures represent the coagulation related genes [THBD, HBEGF, PLAU, ITGAM, ITGB3, PD-ECGF (TYMP)], affected by LASV exposure and the nodes with arrows represent key genes from the immune-response (Akt, ERK, NFkB, p38MAPK and IL1β).

Due to the nature of LF disease, six LASV-upregulated genes related to the coagulation pathway were analyzed (Table 3). Thrombomodulin (THBD), heparin-binding growth factor (HBGF), and integrin alpha M (ITGAM), were up-regulated after LASV exposure at all time-points in contrast to ML29 that down-regulated them. Plasminogen activator urokinase (PLAU) and integrin beta3 (ITGB3) were mostly up-regulated with LASV exposure and remained unchanged or down-regulated with ML29. PD-ECGF (TYMP) was slightly up-regulated after exposure to both viruses. The analysis of those coagulation-related genes using the Gene Ontology (GO) software showed interaction between them and genes related to immune response (Figure 3).

**Table 3:** Coagulation pathway genes affected by Lassa virus.

GenBank accession no.	Name	Gene symbol	Fold changes in gene expression DNA microarray					
			LASV			ML29		
			4 h	8 h	24 h	4 h	8 h	24 h
NM_000361	Thrombomodulin	THBD	1.71	1.26	14.56	1.28	**−1.71**	**−1.07**
NM_001945	Heparin-binding EGF-like growth factor	HBEGF	1.10	4.13	3.80	**−2.45**	**−1.82**	**−1.11**
NM_000632	Integrin, alpha M (complement component 3 receptor 3 subunit)	ITGAM	1.04	1.16	4.36	**−1.12**	**−1.49**	**−1.55**
NM_002658	Plasminogen activator, urokinase	PLAU	1.06	4.79	1.83	**−2.57**	1.20	1.12
NM_000212	Integrin, beta 3 (platelet glycoprotein IIIa, antigen CD61)	ITGB3	1.55	−1.29	12.29	1.79	−1.39	1.48
NM_001953	Endothelial cell growth factor 1 (platelet-derived)	PD-ECGF	−1.10	2.05	3.30	1.04	2.43	1.96

**Figure 4:**
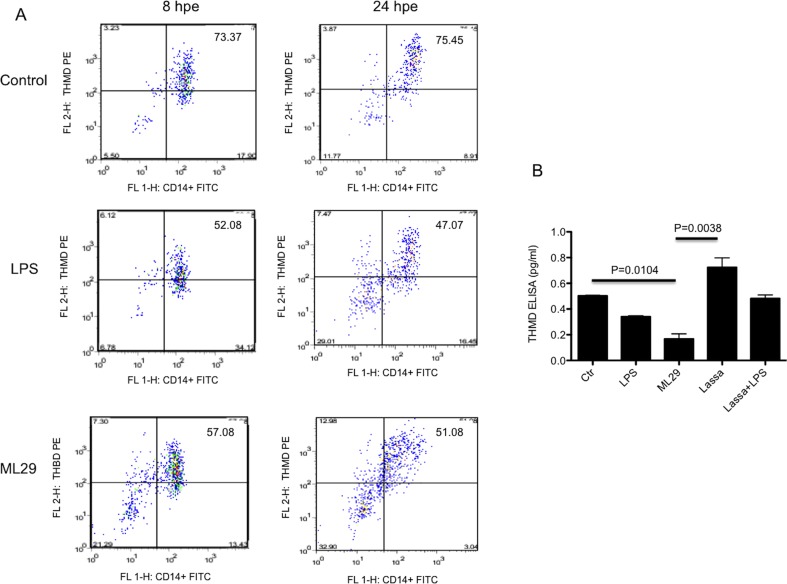
Membrane and soluble thrombomodulin (THBD) measurement. **A**). Cell surface THBD was detected by flow cytometry on gated CD14+ cells. This experiment was repeated with PBMC from 4 different healthy donors with similar results. After exposure to either LPS or ML29, cell-surface THBD was down-regulated by approximately 30%, at both 8 and 24 hours post exposure (hpe). According to a paired Student's t test, this down-regulation was significant (p<0.05) at both time-points. **B**). Soluble THBD measured by ELISA in dendritic cell supernatants exposed to LPS inhibitor, ML29, LASV and LASV plus LPS 24 hpe. Standard deviation of THBD concentrations are shown from three different experiments. THMD from ML29 cells is significantly depressed with respect to THBD from non-exposed control cells. Lassa-exposed cells express significantly more THBD than ML29-exposed cells.

In order to validate cDNA array results, THBD expression was measured on the surface of ML29 exposed-monocytes by flow cytometry, or quantified by ELISA in supernatants of DC exposed to LASV or ML29. There were no significant changes in thrombomodulin expression in CD14+ cells from PBMC treated with LPS or ML29 when compared with non-treated cells at 4 hpe (data not shown). However, as shown in figure 4A, there was a significant reduction in THBD expression on the surface of CD14+ cells after LPS (from ∼74% control to 52% at 8 hpe and 47% at 24 hpe) or ML29 stimulus (from ∼74% control to 57% at 8 hpe and 51% at 24 hpe), all in agreement with microarray results showing lower THBD RNA in ML29-exposed PBMC (Figure 1 and Table S1).

DC cultures exposed to LASV secreted significantly higher levels of THBD than DC exposed to ML29 (Figure 4B), in agreement with transcriptome data. Control DC secreted 0.502 pg/ml THBD, whereas Lassa-exposed DC expressed 0.724 pg/ml THBD, significantly more than ML29-exposed DC (0.161 pg/ml). Treatment with LASV and LPS together diminished the THBD expression from 0.724 pg/ml after LASV exposure to 0.481 pg/ml.

The other coagulation-related genes could also contribute to LF disease: Plasminogen activator urokinase (PLAU) expression could be contributing to the anti-clotting effect mediated by LASV exposure. Heparin-binding epidermal growth factor-like (HB-EGF) expression is involved in several blood vessel physiologies [58], in kidney pathology [59], [60], inhibition of NF-kappa B activation [61], in cardiac hypertrophy, and together with PD-ECGF promotes vascular maturation [62], [63]. The ITGB3 (CD61, platelet marker) was 12-fold up-regulated in cells exposed to LASV at 24 h. Integrins are integral cell-surface proteins composed of α and β chains. A given chain may combine with multiple partners resulting in different integrins. The ITGB3 is found along with the αIIb chain in platelets working as a receptor for fibrinogen, von Willebrand factor, plasminogen, prothrombin, thrombospondin, fibronetin, osteospontin (SPP1), and vitronectin [64], [65], [66]. Integrins are known to participate in cell adhesion as well as cell-surface mediated signaling. High affinity LASV binding to cellular alpha-dystroglycan (α-DG) receptor perturbs the signaling cross-talk between DG and α6β1 integrins, shifting the normal signaling equilibrium towards inhibition of the MEK/ERK pathway [67]. The DG is a ubiquitous receptor for extracellular matrix (ECM) proteins, which cooperates with β1 integrins to control cell-matrix interactions.

## Discussion

When this paper was in post-review, Malhotra et al. published results of transcriptome profiling PBMC from cynomolgous macaques infected with LASV [68]. Their results are in good correlation with the current study and with our previous results in the LCMV-infected monkey model for LF [24] (Table S2).

As would be expected from PBMC exposure to other RNA viruses, transcriptome analysis showed that the set of IFN-stimulated genes (ISG) is the most affected after exposure to LASV or ML29. The antiviral-ISG, including GBP1, IFI44, IFIH1, IFIT1, MX1, and LY6E, were the most common group of up-regulated genes after influenza infection [69], yellow fever vaccination [70], and during febrile episodes of dengue hemorrhagic fever [71], [72].

In comparison to the strong up-regulation by LASV, ML29 has muted expression of INHBA (a negative regulator of IFN-γ), IFI44, TNFSF10 (TRAIL), SPP1, and LY6E (RIG-E) genes in the interferon pathway, as well as other genes related to immune response such as integrin alpha-M/beta-2 (ITGAM). These genes were down-regulated at 4, 8 and, some, 24 hpe, suggesting a mechanism to avoid recognition by the innate immune system, and to interfere with essential signals in the pathway that leads to the development of type I immunity [73], [74], [75]. For example, interferon-alpha/beta inducible IFI44 is involved in the antiviral action of type I interferon [76], [77]. ML29 down-regulation of IFI44 probably allows the virus to replicate early during the infection so that a strong immune response can control viral replication and spreading. In support of this notion, other anti-viral genes such as RSAD2 (Viperin), and TRIM5 [78], [79], [80] were more expressed in LASV than in ML29-exposed PBMC.

LASV up-regulates CXCL6, an IL-8 homolog, as well as three other genes that control cell trafficking: monocyte-attracting chemokines CCL7 [81] and CCL8 (or monocyte chemotactic protein-2, MCP-2 [82] [83], and the mannose receptor C type 1 (MRC1) [84]. These *in vitro* responses may be related to clinical observations of higher serum IL-8 levels in patients with acute non-fatal LF than in control subjects. Levels of IL-8 and other pro-inflammatory cyto/chemokines (IL-1β, IL-6, IL-10, IP-10, TNF-α) were low or undetectable in patients with fatal LF [21] supporting our previous observations in primary human cells [18]. These observations were also confirmed in animal models of fatal LF [20], [17], [85], [30] providing no evidence for the type of “cytokine storm” observed in filovirus HF [86], [87]. The idea that an uncontrolled replication of LASV in targets cells, DC and macrophages, is associated with a marked suppression of innate and adaptive immune responses resulting in clinical progression and death of experimental animals and LF patients is a well-accepted concept.

Bleeding is not a salient manifestation in most cases of LF. In contrast to South American arenaviral HF caused by Junín and Machupo viruses, disseminated intravascular coagulation (DIC) is rarely seen in LF patients. Nevertheless, virus-induced coagulation abnormalities and impaired vascular function is a hallmark of LF pathogenesis [3], [86]. Platelets remain either normal or moderately low, but with significantly reduced function. Plasma from LF patients has inhibitory activity on platelets from healthy individuals, suggesting the presence of soluble factors causing platelets malfunction [88]. Our previous work supports the notion that LASV can replicate vigorously in endothelial cells without cytopathic effects but with reduced cell function, such as reduced production of interleukin IL-8 [18].

Lymphopenia and impaired lymphocyte proliferative responses are also characteristic of severe Lassa fever [89] [88]. Consequently, in addition to hematopoietic effects of soluble factors, lymphocyte apoptosis due to LASV up-regulation of MMP-1 and INHBA may contribute to Lassa pathogenesis.

Coagulation defects associated with LF disease could potentially be affected by LASV-upregulation of six coagulation-related genes, the most notable being thrombomodulin (THBD). THBD is predominantly synthesized by vascular endothelial cells as a 60 kDa type I trans-membrane protein that inhibits thrombotic, inflammatory and redox related responses [90]. THBD forms a 1∶1 molar complex with thrombin and significantly enhances the rate of thrombin inactivation by ATIII while accelerating activation of protein C (aPC). We observed levels of secreted THBD in dendritic cell (DC) culture medium that correlated with our transcriptome data. DC exposure to LASV up-regulated secretion of THBD, whereas exposure to ML29 had the opposite effect. Notably, LPS treatment of LASV-exposed cells resulted in lowering THBD as well. In the presence of thrombin, THBD generates aPC, which inhibits pro-coagulant and pro-inflammatory responses including: fibrinogen cleavage, factor V activation, platelet activation or local cytokine-induced chemotaxis for monocytes, neutrophils and up-regulation of leukocyte adhesion molecules [91], [92], [93]. It has been shown that THBD mRNA half-life is shortened by treatment of cells with IFN-γ [94]. This observation in addition to the LASV-increased THBD expression seen here, and the absence of IFN responses seen *in vitro*, are in agreement with LF findings where there is no clear inflammatory response and no cellular infiltrates, in conjunction with the high viral loads seen in the presence of hemorrhagic symptoms. The absence of THBD up-regulation in the LCMV-WE infected model for LF may be a key failing of the model (see details in Table S2).

**Figure 5:**
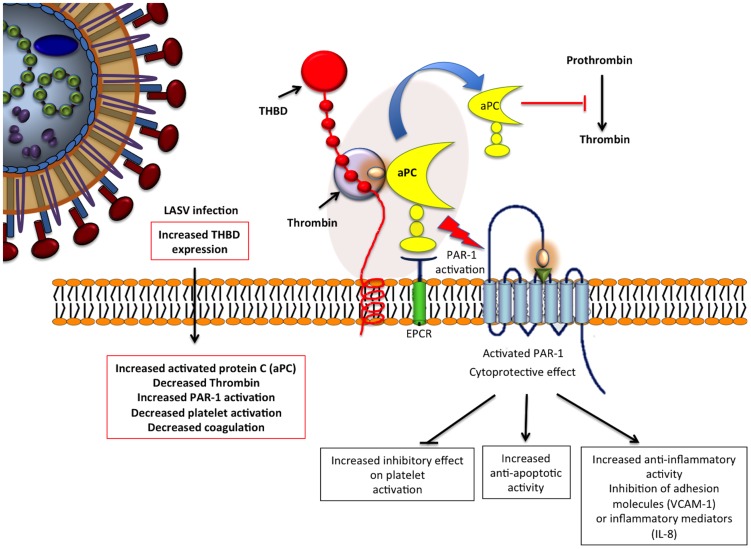
Thrombomodulin (THBD) in LASV infection. LASV infects endothelial cells, which are the main THBD producers. Increased THBD expression will capture thrombin through its 4, 5 and 6 domains, activating protein C (aPC), inhibiting the coagulation pathway and activating PAR1 membrane protein. PAR-1 has cytoprotective and anti-inflammatory affects, also inhibiting platelet activation.

Our current hypothesis is that LASV infects endothelial and antigen-presenting cells and increases the expression of both membrane and soluble THBD forms, activating aPC or capturing HMGB1 with anti-inflammatory and anti-coagulation effects (Figure 5). The up-regulation of THBD and its consequent anti-coagulation effects could also explain why fibrin deposits are rarely seen in histological examination of tissues from LF patients and disseminated intravascular coagulation (DIC) is rare in LF, but frequently observed in other viral hemorrhagic fevers. Given these results, inhibition of THBD could be a potential therapeutic intervention for LF.

LASV-induced perturbation of the cross-talk between integrins and the extracellular matrix [67] may contribute to the functional alterations of epithelial and vascular endothelial cells that precede shock and death in LF patients [95]. It is also possible that, during high states of viremia, LASV binds directly to platelets inhibiting their function. After an arterial injury, leukocytes are recruited to the site and there is an increase in ITGAM expression allowing adhesion of platelets and fibrinogen to the vessel walls [96]. We propose that LASV induction of ITGAM could increase platelet adhesion to tissues and this may explain the decreased number of circulating platelets during hemorrhagic episodes. Platelet-derived endothelial cell growth factor (PD-ECGF) is involved in angiogenesis and wound repair [97], [98]. The differential expression of all those genes may be contributing to the non-pathogenic phenotype of ML29 and to the pathogenic phenotype of LASV.

In LASV-infected primates, DC and macrophages were identified as prominent targets at early stages of infection, while Kupffer cells, hepatocytes, adrenal cortical cells, and endothelial cells were more frequently infected at late stages [99]. We showed previously that mononuclear cells in PBMC are not permissive for LASV and the virus can replicate only in differentiated monocytes, DC and macrophages [18], representing less than 1% of circulating PBMC. Since LASV and ML29 replicate at the same rate in monocyte-derived cultures (ISL unpublished), the differential gene expression reported here is most likely due to differential signaling of uninfected bystander cells.

Both viruses, LASV and ML29, have identical surface-exposed GP1 responsible for interaction with α-DG; however ML29 and LASV GP2 differ by the lysine to glutamic acid (K272E) substitution located between the fusion domain and the RRLL motif, the cellular subtilase SKI1/S1P cleavage site [26] [7]. The amount of negative charge at that site in ML29 is greater than in other arenaviral GP2, and could potentially affect fusion and signaling by ML29.

In summary, exposure of human PBMC to viruses modeled the viremic stage of disease, and revealed clear differences between responses to LASV and the attenuated ML29. It remains to be shown whether the charge difference in LASV and ML29 GP2 proteins accounts for their different transcriptome profiles. ISG and genes involved in apoptotic and NF-kB pathways were among the most LASV-affected genes at 24 h after exposure. LASV and not ML29 also affected genes involved in coagulation pathways, e.g. LASV strongly up-regulates THBD suggesting a connection to the vascular abnormalities in fatal cases of LF. Based on our current knowledge about THBD involvement in pro-coagulant and pro-inflammatory pathways, THBD inhibition could be a promising drug target. Additionally, it is important to further investigate the immune response-related genes to elucidate the protective mechanisms that are undermined during LF pathogenesis.

## Supporting Information

Figure S1 Cluster analysis showed that all samples clustered together by time (4, 8, and 24 hpe), by donor (3 healthy donors) and by treatment (unexposed, LASV-exposed, and ML29-exposed cells). Sample IDs are organized by Time-Donor-Stimulus-Sample number. Red color represents up-regulated genes and green color represents down-regulated genes.
(TIF)

Table S1 A total of 122 genes were identified as being differentially expressed at tested time-points. 136 appear in the table due to the use of more than one probe to detect the same gene. Column A shows the Affymetrix gene name. Column B corresponds to the probe identification reference. Columns C to E are the comparison between LASV exposure and un-exposed cells at different time points. Columns F to H are the comparison between ML29 and un-exposed cells at different time points. Column I has the Unigene ID numbers, and column J has the GenBank accession numbers. The numbers indicate fold-changes in gene expression when compared with the control. Red color represents the more significant up-regulated genes. Green color represents the more significant down-regulated genes.
(TIF)

Table S2 SAM ANALYSIS of the RNA profiles of PBMC exposed to LASV and ML29 *in vitro* compared to RNA profiles of PBMC from WE/ARM infected monkeys. ∼140 genes with common patterns and ∼7 with different patterns, all at 24 hpe. The navy blue highlighting indicates the discrepancies in transcriptome between the LASV/ML29 pair and the LCMV-WE/ARM pair. Yellow highlighting indicates interferon pathway genes and coagulation-related genes.
(TIF)
