# Supplementary figures and images for: Transcriptome Analysis of Human Peripheral Blood Mononuclear Cells Exposed to Lassa Virus and to the Attenuated Mopeia/Lassa Reassortant 29 (ML29), a Vaccine Candidate
Source: PLoS Negl Trop Dis. 2013 Sep 12;7(9):e2406. doi: 10.1371/journal.pntd.0002406 (PMC3772037; doi:10.1371/journal.pntd.0002406)

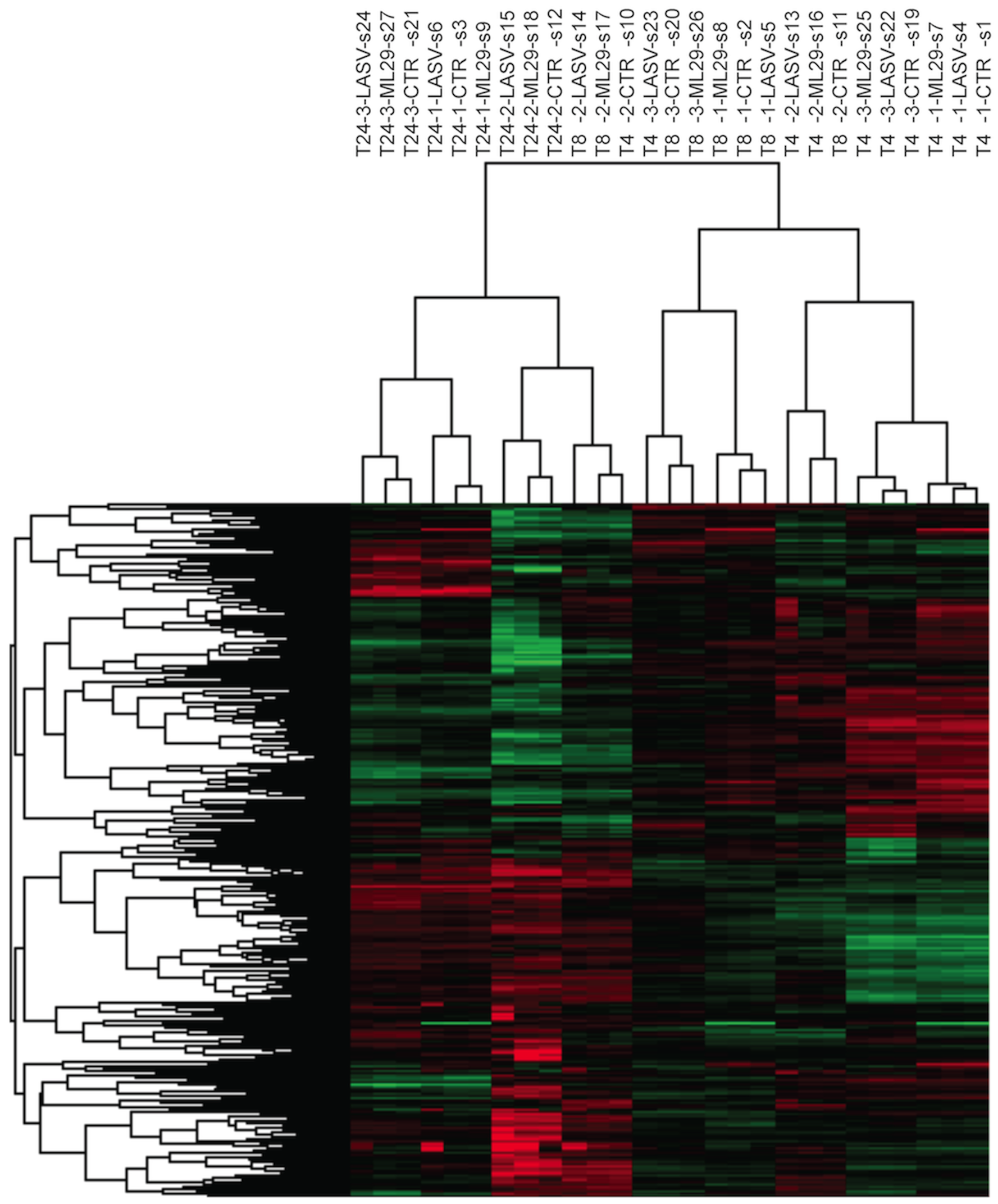

Supplement: Figure S1 — Cluster analysis showed that all samples clustered together by time (4, 8, and 24 hpe), by donor (3 healthy donors) and by treatment (unexposed, LASV-exposed, and ML29-exposed cells). Sample IDs are organized by Time-Donor-Stimulus-Sample number. Red color represents up-regulated genes and green color represents down-regulated genes. (TIF) [file pntd.0002406.s001.tif]

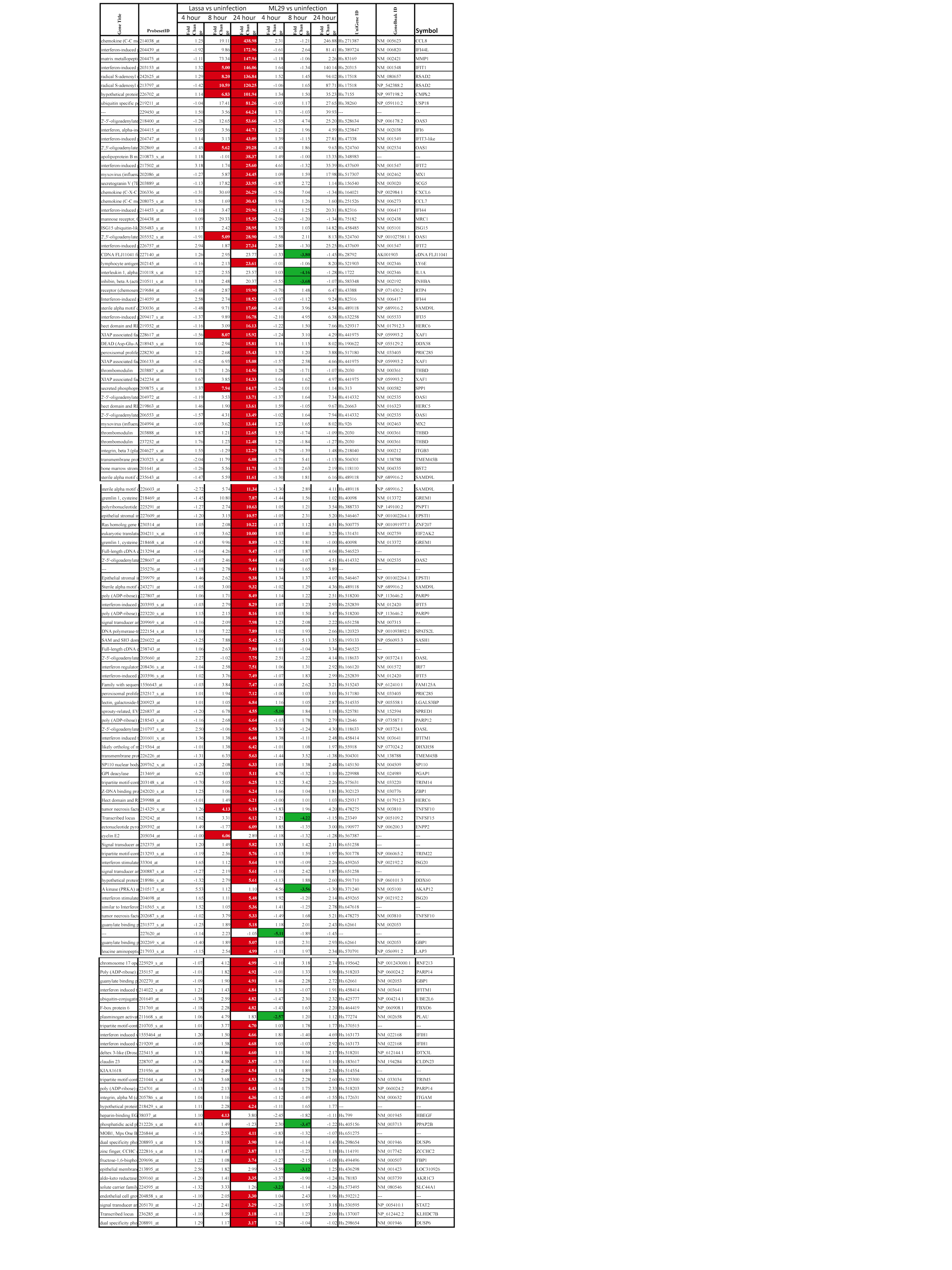

Supplement: Table S1 — A total of 122 genes were identified as being differentially expressed at tested time-points. 136 appear in the table due to the use of more than one probe to detect the same gene. Column A shows the Affymetrix gene name. Column B corresponds to the probe identification reference. Columns C to E are the comparison between LASV exposure and un-exposed cells at different time points. Columns F to H are the comparison between ML29 and un-exposed cells at different time points. Column I has the Unigene ID numbers, and column J has the GenBank accession numbers. The numbers indicate fold-changes in gene expression when compared with the control. Red color represents the more significant up-regulated genes. Green color represents the more significant down-regulated genes. (TIF) [file pntd.0002406.s002.tif]

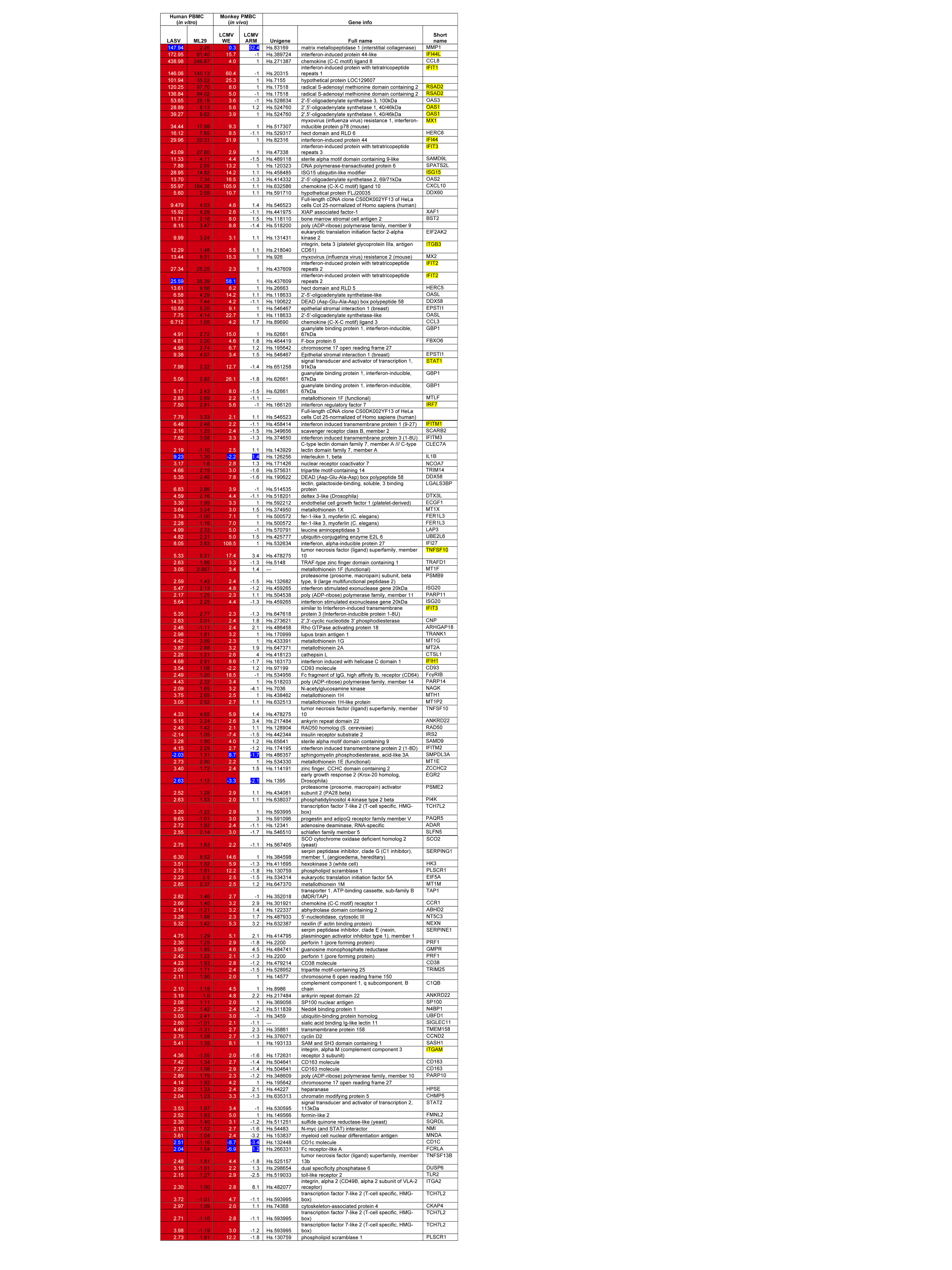

Supplement: Table S2 — SAM ANALYSIS of the RNA profiles of PBMC exposed to LASV and ML29 in vitro compared to RNA profiles of PBMC from WE/ARM infected monkeys. ∼140 genes with common patterns and ∼7 with different patterns, all at 24 hpe. The navy blue highlighting indicates the discrepancies in transcriptome between the LASV/ML29 pair and the LCMV-WE/ARM pair. Yellow highlighting indicates interferon pathway genes and coagulation-related genes. (TIF) [file pntd.0002406.s003.tif]
